# An Update on the Genus *Aeromonas*: Taxonomy, Epidemiology, and Pathogenicity

**DOI:** 10.3390/microorganisms8010129

**Published:** 2020-01-17

**Authors:** Ana Fernández-Bravo, Maria José Figueras

**Affiliations:** Unit of Microbiology, Department of Basic Health Sciences, Faculty of Medicine and Health Sciences, IISPV, University Rovira i Virgili, 43201 Reus, Spain; ana.fernandez@urv.cat

**Keywords:** *Aeromonas*, taxonomy, epidemiology, immune response

## Abstract

The genus *Aeromonas* belongs to the *Aeromonadaceae* family and comprises a group of Gram-negative bacteria widely distributed in aquatic environments, with some species able to cause disease in humans, fish, and other aquatic animals. However, bacteria of this genus are isolated from many other habitats, environments, and food products. The taxonomy of this genus is complex when phenotypic identification methods are used because such methods might not correctly identify all the species. On the other hand, molecular methods have proven very reliable, such as using the sequences of concatenated housekeeping genes like *gyrB* and *rpoD* or comparing the genomes with the type strains using a genomic index, such as the average nucleotide identity (ANI) or *in silico* DNA–DNA hybridization (*is*DDH). So far, 36 species have been described in the genus *Aeromonas* of which at least 19 are considered emerging pathogens to humans, causing a broad spectrum of infections. Having said that, when classifying 1852 strains that have been reported in various recent clinical cases, 95.4% were identified as only four species: *Aeromonas caviae* (37.26%), *Aeromonas dhakensis* (23.49%), *Aeromonas veronii* (21.54%), and *Aeromonas hydrophila* (13.07%). Since aeromonads were first associated with human disease, gastroenteritis, bacteremia, and wound infections have dominated. The literature shows that the pathogenic potential of *Aeromonas* is considered multifactorial and the presence of several virulence factors allows these bacteria to adhere, invade, and destroy the host cells, overcoming the immune host response. Based on current information about the ecology, epidemiology, and pathogenicity of the genus *Aeromonas*, we should assume that the infections these bacteria produce will remain a great health problem in the future. The ubiquitous distribution of these bacteria and the increasing elderly population, to whom these bacteria are an opportunistic pathogen, will facilitate this problem. In addition, using data from outbreak studies, it has been recognized that in cases of diarrhea, the infective dose of *Aeromonas* is relatively low. These poorly known bacteria should therefore be considered similarly as enteropathogens like *Salmonella* and *Campylobacter*.

## 1. Introduction

Based on the most recent edition of the Bergey’s Manual [1], the genus *Aeromonas* (aer-, from Greek: gas; -monas: units; i.e., gas-producing units), belongs to the class of Gammaproteobacterias, order Aeromonadales, and family *Aeromonadaceae*, embracing three genera: *Aeromonas*, *Oceanimonas*, and *Tolumonas* [1]. The members of this genus are characterized as Gram-negative bacilli (0.3–1.0 × 1.0–3.5 µm), oxidase and catalase positive, capable of degrading nitrates to nitrites, glucose fermenters and resistant with few exceptions [2] to vibriostatic factor O/129 (2,4-Diamino-6,7-di-iso-propylpteridine phosphate). This genus comprises 36 species that are considered autochthonous of aquatic environments. They are also isolated from foods, animals, and various infectious processes in humans [1,3,4,5,6].

The taxonomy of the genus *Aeromonas* has always been subject to change, with 22 new species added since 1992. Identification to the species level using phenotypical characterization is difficult due to the variable behavior of the strains, which has caused a lot of confusion and erroneous identifications [7]. Molecular identification methods such as the sequences of housekeeping genes and their concatenated phylogenetic analysis (i.e., multilocus phylogenetic analysis (MLPA)) have helped to correctly recognize many of the new species of the genus by overcoming the limitation of the high similarity of the 16S rRNA gene that exits among some closely related species [8,9,10,11]. In addition, the development of several tools for genome comparison and characterization has enabled analysts to recognize wrongly labeled genomes and the role of the bacteria in the environment [12,13,14].

Several species of *Aeromonas* are considered as emerging pathogens because they caused a wide spectrum of disease in humans, mainly gastroenteritis, wound infections, and bacteremia/septicemia, infecting immunocompromised and immunocompetent [3,4,5]. Studies have so far reported that 96.5% of the strains associated to clinical cases were identified as one of only four species: *Aeromonas caviae* (29.9%), *Aeromonas dhakensis* (26.3%), *Aeromonas veronii* (24.8%), and *Aeromonas hydrophila* (15.5%) [5,15]. In addition, other species usually linked with fish diseases, such as *Aeromonas salmonicida*, have also been reported in human infections [16]. Moreover, one important issue in the study of these infections is the selection of an adequate animal model or cell line that would reproduce the pathogenicity of *Aeromonas* [17].

The virulence of *Aeromonas* has been described as multifactorial and linked to the expression of genes (*exoA*, *alt*, *act*, etc.) that encode different toxins, structural components (*flaA*, *maf-5*, *flp*, etc.*)*, secretion systems (T3SS, T6SS, etc.), and proteins associated with metals [18,19]. The presence of these virulence factors allows the bacteria to colonize, invade, and overcome the immune response mechanism of the host, resulting in an infection that generates the disease [4,5,18,20]. Several studies demonstrated the expression of different immune-related genes in the host following an *Aeromonas* infection, including those involved in pathogen recognition, the proteins involved in cell signaling, and apoptosis [21,22,23,24].

The aim of this review is to provide an update on the genus *Aeromonas*, including recently acquired knowledge of the taxonomy, ecology, epidemiology and pathogenicity of the bacteria of this genus.

## 2. Historic Perspective: Past and Present

The first isolates, thought to date back to 1891, were reported by Sanarelli et al. [25], who named the bacteria as *Bacillus hydrophilus fuscus* (now *Aeromonas hydrophila).* However, the International Committee of Systematic Bacteriology established the authorship of the genus to Stanier in 1943. This genus was included in the family *Vibrionaceae* in 1965, with the genera *Vibrio* and *Plesiomonas*. In 1986, Colwell et al. [26] carried out a sequence analysis of the 16S rRNA and 5S rRNA genes and DNA–DNA hybridization studies, and demonstrated that *Aeromonas* formed a different phylogenetic branch, thus creating the *Aeromonadaceae* family. In the mid-1970s, the species of the genus were divided into two groups based on different characteristics: mesophilic group (optimal growth at 35–37 °C) with motile isolates, responsible for several infections in humans and defined as “*A. hydrophila*” and a psychrophilic group (optimal growth 22–28 °C) with non-motile strains, linked to fish diseases and identified under “*A. salmonicida*”. However, in the last years, many studies suggested that a growth temperature greater than 22 °C induces major rearrangements in *A. salmonicida*, such as the loss of the type three secretion system (T3SS) which is an important virulence factor in the bacteria, resulting in non-virulent strains in fish and model hosts. This is consistent with the fact that this bacterium infects cold-water fish, including salmonids that do not survive in temperatures above 25 °C [27,28].

**Figure 1:**
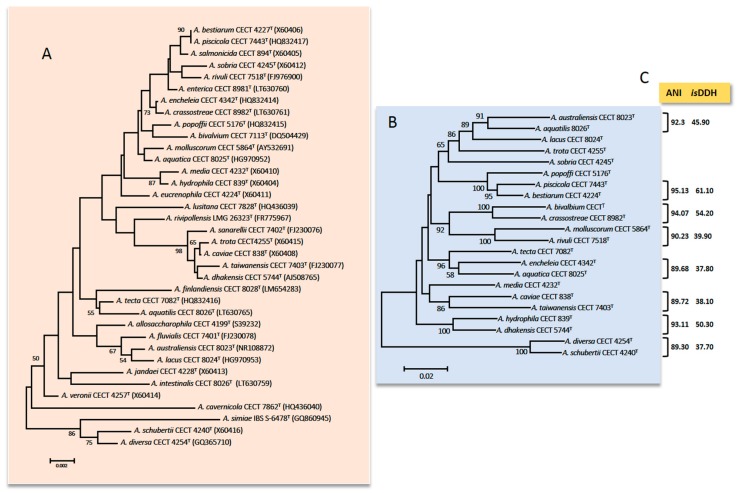
(**A**) Phylogenetic tree based on the sequences of the 16S rRNA gene (1498 bp) among 36 species of *Aeromonas.* (**B**) Phylogenetic tree based on the concatenated sequences of *rpoD* and *gyrB* genes (1098) among the most similar species based on the 16S rRNA gene. The number in the nodes indicates the bootstrap values substitutions estimated by site. (**C**) Results (%) for the ANI (average nucleotide identity) and *is*DDH (*in silico* DNA–DNA hybridization) obtained between the genomes of the most similar species; notice that ANI and *is*DDH values are ≤96% and ≤70% in all cases, respectively which are the cut-off values established for delimiting *Aeromonas* spp.

The genus currently comprises 36 species (Figure 1) that have been described since 1943: *Aeromonas allosaccharophila* [29], *Aeromonas aquatica* [30], *Aeromonas aquatilis* [31], *Aeromonas australiensis* [32], *Aeromonas bestiarum* [33], *Aeromonas bivalvium* [34], *Aeromonas cavernicola* [35], *A. caviae* [36], *Aeromonas crassostreae* [31], *A. dhakensis* [37], *Aeromonas diversa* [38], *Aeromonas encheleia* [39], *Aeromonas enterica* [31], *Aeromonas eucrenophila* [36], *Aeromonas finlandiensis* [30], *Aeromonas fluvialis* [40], *A. hydrophila* [41], *Aeromonas intestinalis* [31], *Aeromonas jandaei* [42], *Aeromonas media* [43], *Aeromonas molluscorum* [44], *Aeromonas lacus*, *Aeromonas lusitana* [2], *Aeromonas piscicola* [45], *Aeromonas popoffii* [46], *Aeromonas rivipollensis* [47], *Aeromonas rivuli* [48], *A. salmonicida* [49], *Aeromonas sanarellii* [50], *Aeromonas schubertii* [51], *Aeromonas simiae* [52], *Aeromonas sobria* [53], *Aeromonas taiwanensis*, *Aeromonas tecta* [54], *Aeromonas trota* [42], and *A. veronii* [55].

## 3. Taxonomy and Identification Methods

For the description of new prokaryotic species, the International Committee on Systematics of Prokaryotes (ICSP) recommends a polyphasic study, which should include phenotypic and phylogenetic differentiation from existing species [56]. A discussion of the criteria proposed by the ICSP in relation to the genus *Aeromonas* is given elsewhere [57].

### 3.1. Phenotypic Identification

Phenotypic identification is made by physiological, morphological, and biochemical characteristics [4,56,58]. Classic phenotypic characteristics that identify the genus *Aeromonas* are Gram-negative staining, the presence of normally positive cytochrome oxidase, and growth in nutritive broth at 0% to grow in the presence of vibriostatic factor O/129 [4,58]. Despite that, identification to the species level using this approach is difficult due to the variable behavior of the strains. In 2010, Beaz-Hidalgo et al. [7] re-identified 119 strains, isolated mainly from diseased fish that had previously been identified phenotypically. The re-identification was carried out by molecular methods (16S rRNA-RFLP and *rpoD* sequences) and the results demonstrated that only 35.5% were correctly identified at the species level.

Additionally, commercial identification systems (API 20E, Vitek, BBL Crystal, MicroScan W/A, among others) have commonly been used in clinical laboratories, although several authors demonstrated that these systems had limitations [10,59]. In 2010, Lamy et al. [59] compared the accuracy of six commercial systems for *Aeromonas* identification, using the *rpoB* sequencing as a reference. Concordance was shown to be low between phylogenetic identification and the commercial identifications systems, with erroneous identification at species level. The study also ratified results of previous studies that highlighted the confusion between *Aeromonas* and the genus *Vibrio* [59,60,61]. This confusion was evidenced again in a clinical case report that identified the strain involved as *Vibrio alginolyticus*, while the preliminary identification at the hospital corresponded to *Aeromonas* spp. using API20E [20].

### 3.2. Molecular Identification

#### 3.2.1. Techniques Based on the 16S rRNA Gene

The 16S rRNA gene is considered a stable molecular marker for identifying bacterial species, since its distribution is universal and allows comparison of microorganisms [56,57,62,63]. In addition, its structure presents a mosaic of variable regions, suitable in the differentiation of closely related organisms, and their conserved regions are useful for the distant organisms comparison and this allows for the design of “universal” primers [64].

In 1992, Martínez-Murcia et al. [8] sequenced the 16S rRNA gene for the first time using strains of the species described up to then; the results agreed with the DNA–DNA hybridization (DDH). In the genus *Aeromonas*, the 16S rRNA gene has an interspecies similarity range from 96.7–100% and the informative nucleotide positions are located mainly on region V3 [63,64]. 

Additionally, the presence of microheterogeneities (i.e., mutations on specific positions of the sequence of one of several copies of the 16S rRNA gene) in combination with the high similarity of the sequences for closely related species makes this gene not suitable for the *Aeromonas* spp. identification [64,65,66]. Figure 1 shows the phylogenetic tree derived from sequences of the 16S rRNA gene of the 36 *Aeromonas* species.

Matrix-assisted laser desorption/ionization time of flight mass spectrometry (MALDI-TOF MS) is a powerful method introduced to many clinical laboratories in recent years for the identification and comparison of microbial isolates [20,67]. The MALDI-TOF MS mainly detects proteins associated with the 16S rRNA gene and therefore the low resolution of this gene for the identification of closely related species of *Aeromonas* also impacts the resolution of this method [15].

Chen et al. [68] used the MALDI-TOF MS to characterize 217 clinical isolates previously identified by *rpoB* sequencing and found that 100% were correctly identified at genus level, and 97% at species level. One-year later, Shin et al. [69] re-identified 65 clinical strains previously identified by *gyrB* sequencing and showed 98.5% concordance at genus level, and 92.3% at the species level using the MALDI-TOF MS. These results are relatively similar to those reported by Latif-Eugenín [15] who identified 179 clinical strains from Spanish hospitals, with 98.3% correct identification at genus level, and 91.1% at species level using MALDI-TOF MS. Based on those data, they suggested that MALDI-TOF MS is a useful tool, since the identification error was <10%, while with phenotypic identification methods error can be very high. The main limitation of the latter method is the need to update the database to include the many missing *Aeromonas* species, such as *A. dhakensis* or the new species (*A. intestinalis*, *A. crassostreae*, *A. enterica*, and *A. aquatilis*). A recent study that used MALDI-TOF MS for the characterization of *Aeromonas* strains isolated from fish [70] demonstrated that the number of correct identifications increased after the addition of 14 new spectra in the MALDI-TOF Biotyper database.

#### 3.2.2. Housekeeping Genes

Housekeeping genes (HKG) encode proteins with essential functions for the survival of bacteria. They were introduced for the description of new species using an MLPA because the resolution is higher than the 16S rRNA gene [56,71]. For taxonomic analysis, the ideal HKG should have the following characteristics: (1) they should not be influenced by horizontal gene transfer; (2) they should be present in all bacteria; (3) they should be single genes in the genome of the bacteria; (4) and finally they should present at least two conserved regions for the design of primers [72].

The first HKG studied of *Aeromonas* was the *gyrB* gene that encodes the subunit B of DNA gyrase [9]. Another HKG that shows a similar phylogeny to *gyrB* is the *rpoD* gene that encodes sigma factor S70 (that confers promoter-specific transcription initiation for the RNA polymerase) [10]. These genes were used to recognize and describe many species in recent years [2,30,31,32,35,37,38,40,45,47,48,50,54]. The phylogenetic tree derived from sequences of *gyrB* and *rpoD* genes of 22 highly similar *Aeromonas* species based on the 16S rRNA gene is presented in Figure 1. Many studies have described other HKG: *rpoB*, *recA*, *dnaJ*, *cpn60*, *mdh*, *gyrA*, *dnaX*, *atpD*, *groL*, *gltA*, *metG*, *ppsA*, *dnaK*, *radA*, *tsF*, and *zipA* [11,38,66,73,74,75,76,77]. However, the phylogeny based on the sequence of only one HKG is sometimes not conclusive and a higher resolution is obtained using the concatenated sequences of several HKG [56,57,64]. In 2011, Martínez-Murcia et al. [11] described the first MLPA of the genus *Aeromonas* using the concatenated sequences of seven genes (*rpoD*, *gyrB*, *gyrA*, *atpD*, *recD*, *dnaJ*, and *dnaX*).

#### 3.2.3. Genotyping Methods

Different molecular methods have been employed to trace whether two isolates of *Aeromonas* belong, or not, to the same clone and therefore share an epidemiological relationship [20,64]. These methods are the enterobacterial repetitive intergenic consensus-PCR (ERIC-PCR), the randomly amplified polymorphic DNA-PCR (RAPD-PCR), the amplified fragment length polymorphism (AFLP), the pulsed-field gel electrophoresis (PFGE), and the multilocus sequence typing (MLST) [20,64].

The ERIC-PCR is one of the most popular methods for genotyping *Aeromonas* because it is easy to carry out, does not require any expensive equipment, and is highly reproducible [3,78]. Consequently, it has been used in several epidemiological studies [79,80,81,82]. In a recent study, one strain of *A. caviae* isolated from a sample of lettuce showed the same ERIC genotype pattern as a strain recovered from a sample of irrigation water [82]. In addition, the same genotype of *A. sanarellii* was recovered in samples of parsley and tomato that were irrigated with the same reclaimed water, confirming the potential health risk to humans [82].

The MLST is based on the analysis of the sequences of several genes, normally seven, to recognize allele sequences (83). This technique show to be highly discriminatory and reproducible compared with other techniques, and there is also a database to help investigators compare their results. The Bacterial Isolate Genome Sequence Database (BIGSdb) is the platform that currently manages the MLST database [83] and can be found within the PubMLST public databases. The MLST scheme is freely available and was created for *Aeromonas* in 2010 based on the data obtained by Martino et al. [77] using six genes (*gyrB*, *groL*, *gltA*, *metG*, *ppsA*, and *recA*). The major problem of the latter technique is the need for perfect sequences of seven housekeeping genes (450–500 bp each gene) with no ambiguities, and result comparison might be limited by the number of strains and origins available in the database, which in its last update (22 October 2019) had 751 strains, and 2817 sequences that corresponded to 652 MLST profiles (available online: https://pubmlst.org/Aeromonas/submission.shtml consulted on 22 October 2019).

#### 3.2.4. Genomics

As of 2 September 2019, 410 *Aeromonas* genomes were made publicly available in the GenBank database, of which 63 are complete (available online: https://www.ncbi.nlm.nih.gov/genome/?term=Aeromonas). The size of *Aeromonas* genomes varies between 3.90 Mbp (*A. fluvialis*) and 5.18 Mbp (*A. piscicola*) with an average size of 4.51 Mbp [19]. Furthermore, the percentage of G + C was 60.2%, varying between 58.1% (*A. australiensis*) and 62.8% (*A. taiwanensis*).

Advances in methods of obtaining complete genomes have increased the number of available genomes in recent years. In fact, only six *Aeromonas* genomes were available in 2012 [84,85,86,87,88,89] and just two years later, that increased to 56 genomes representing 29 recognized or proposed species of the genus *Aeromonas* [90]. In 2015, using MLPA and pairwise comparison using the average nucleotide identity (ANI), Beaz-Hidalgo et al. [12] re-identified 44 genomes that were deposited in GenBank, demonstrating that 14 were wrongly labeled by using the MLPA and pairwise comparison using the ANI. The data obtained in that study showed the importance of verifying the taxonomic position of a genome, using the mentioned tools (MLPA and ANI) before submission to the NCBI or other databases. These misidentifications might also be determined using another tool based on genome comparison (i.e., *in silico* DNA–DNA hybridization (*is*DDH)) [12,58,90].

The experimental DDH is commonly used for species delineation [48]. However, this technique produces errors and takes up a lot of time. The *is*DDH described by Kolthoff et al. [91], using the genome-to-genome distance calculator (GGDC) developed by DSMZ (Leibniz Institute DSMZ-German Collection of Microorganisms and Cell Cultures GmbH, Braunschweig, Germany) showed to be an excellent tool for determining the genetic similarity between two bacteria genomes. The results ≥70% indicates that these two strains belong to the same species (Figure 1). Moreover, in 2009, Richter and Rosselló-Mora [92] defined the ANI as the percentage of identity that can be found in the nucleotide sequences of orthologous genes common in the two genomes (Figure 1). Based on different studies [92,93] the cut-off value was established at 95–96% and the results agreed with *is*DDH. There are several tools to calculate ANI values: JSpecies, ANI calculator, OrthoANI, and OrthoANI-usearch tool. The cut-off for *Aeromonas* was established in 2014 by Colston et al. [90]. In the study, 56 *Aeromonas* genomes were analyzed, suggesting that values ≥96% indicate that the two strains belong to different species. Figueras et al. [14] indicated that the ANI and the MLPA are excellent tools for verifying the identity of genomes before they are deposited in GenBank, which would prevent them from being mislabeled. In fact, Beaz-Hidalgo et al. [12] used these tools for re-identifying the genomes deposited in the NCBI, and found that 35.9% of the genomes of non-type strains of *Aeromonas* spp. were incorrectly labeled. Recently, different studies used the genomic indices to increase the correct identification of ambiguous *Aeromonas* strains, supporting the notion that these methods are essential in taxonomy [94,95,96].

## 4. Ecology and Epidemiology

The genus *Aeromonas* is widely distributed across numerous ecosystems, although it is more commonly found in various aquatic environments [4,6,32]. These microorganisms have also been isolated from several environmental and clinical samples [4,97].

### 4.1. Aeromonas in Aquatic Environments

*Aeromonas* are indigenous to aquatic environments and have been isolated from surface, underground, potable, bottled, residual, seawater, and irrigation waters [4,6,98,99,100,101,102,103]. Araujo et al. [104] established that the concentration of *Aeromonas* can be linked to terrestrial water effluents. In a study of rivers and lakes in Finland, 116 strains of *Aeromonas* were recovered, which were thought to be linked to cyanobacteria blooms [105,106] and these strains were re-identified by Beaz-Hidalgo et al. [5], thus allowing the description of three new species (*A. aquatica*, *A. finlandiensis*, and *A. lacus*).

The incidence of *Aeromonas* in wastewater is high [107,108,109,110,111]. Conventional treatments to reclaim water at wastewater treatment plants (WWTPs), such as a primary and secondary or biological treatment, do not greatly reduce the concentration of *Aeromonas* [6]. However, if additional tertiary treatments are carried out such as chemical (ozone, chlorination) and physical (ultraviolet radiation) treatments, *Aeromonas* can be completely eliminated [82,112,113,114]. Another natural tertiary treatment is lagooning. It is a process of purification which reduces the levels of *Aeromonas* by temporarily storing the wastewater in shallow ponds or lagoons [115,116,117]. Depending on the microbiological quality of the reclaimed water, it can be used for irrigation [32,82,113]. Irrigated waters containing *Aeromonas* can contaminate fruits and vegetables [82,118] and thus cause infection in humans. In fact, there is epidemiological evidence that water acts as a vehicle for the dissemination of this bacterium, because it has been possible to identify the same *Aeromonas* strains in drinking water samples and in the feces of individuals with diarrhea [119,120]. In another study, the same strains isolated from lettuce and tomatoes were recovered from the irrigation water [113].

There are numerous studies in which the presence of *Aeromonas* was detected in drinking water [3,6,100] and concentrations are related to the ability to regrow in the system [110,121], which is influenced by the amount of available nutrients, temperature, and concentration of residual chlorine [6,122]. Latif-Eugenín et al. [82] also showed the importance of culture conditions for the detection of *Aeromonas* in water.

Metagenomic studies of wastewater revealed *Aeromonas* to be one of the prevailing bacteria probably due to its capacity to regrow in the sewerage system [107].

### 4.2. Aeromonas in Food

*Aeromonas* have been isolated from fruits, vegetables, dairy products, meats and sausages, fish, and shellfish [58,123,124,125,126,127,128,129]. The temperature, salinity or pH are the factors that determine the number of *Aeromonas* in these foods [1]. In fact, in the case of temperature, several authors reported that *Aeromonas* survive in low temperatures (2–10 °C). Additionally, salt or sodium chloride (NaCl) is a common preservative for meat products and raw fish, however, *Aeromonas* are able to grow in NaCl concentrations of up to 4%. Finally, pH is another parameter that controls bacterial growth in food. It was demonstrated that *Aeromonas* can survive at pH = 5 [14,130].

Many studies have investigated the incidence of *Aeromonas* in meat products [128,131,132]. Based on the sequencing of HKG like *rpoD* and *gyrB*, the most prevalent species were *A. veronii*, *A. salmonicida*, *A. jandaei A. simiae*, *A. hydrophila*, and *A. caviae* [81,125]. In the case of fish intended for human consumption, tilapia and salmonids bare many *Aeromonas* spp. that after molecular identification corresponded to *A. salmonicida*, *A. bestiarum*, *A. veronii*, *A. encheleia*, *A. hydrophila*, *A. allosaccharophila*, and *A. bivalvium* [128,133]. In relation to shellfish, *Aeromonas* is often isolated from mussels, oysters, shrimps, and cockles, and based on molecular identification methods the diversity of *Aeromonas* species is similar to the species found in fish [128,134,135]. However, as several authors reported, *Aeromonas* infections are often under-diagnosed or even misdiagnosed, changing the values based on the diagnostic methods.

Different studies report the isolation of *Aeromonas* from fruits and vegetables, such as carrots, tomatoes, lettuces, cucumber, potatoes, onions, and celery [123,124,125]. Among the species detected *A. hydrophila* and *A. caviae* were the most prevalent clinical species [123,124,125]. These foods, together with contaminated water are the main source of infection by *Aeromonas* in diarrhea [128,136].

### 4.3. Aeromonas in Animals

The genus *Aeromonas* has been considered an animal pathogen since its first isolation from septicemias in frogs and sick fish [7,45,137]. The important fish pathogens species are *A. salmonicida* and *A. hydrophila*, which particularly affect salmonids, causing ulcers, hemorrhages, furunculosis, and septicemias [7,45]. These infections cause important financial losses in the aquaculture industry [4,7,16,45,88]. In fact, Rasmussen-Ivey et al. [138] described a hypervirulent *A. hydrophila* strain as a causative agent of worldwide outbreaks in warm-water fishes. Several studies have isolated other species from fish: *A. veronii* from catfish [139]; *A. piscicola* from salmonids [7,45]; *A. sobria* from tilapia [140]; *A. schubertii* from snakehead fish [141]; *A. veronii*, *A. bestiarum*, *A. encheleia*, and *A. sobria* from common carp; and *A. allosaccharophila*, *A. dhakensis*, *A. caviae*, *A. veronii*, *A. hydrophila*, *A. jandaei*, *A. media*, and *A. trota* from eel [29,39,142,143,144].

*Aeromonas* were isolated following outbreaks in rabbit farms as well as recovered from the feces of dogs, cats, and horses [145,146,147,148]. Several publications described *Aeromonas* as a pathogen of echinoderms, mollusks, and crocodiles and associated with copepods [34,44,149,150].

### 4.4. Aeromonas in Human Infections

*Aeromonas* are an emerging pathogen that cause a wide range of diseases in humans, commonly gastroenteritis, septicemia, and wound infections, and are able to infect both immunocompromised and immunocompetent patients [4,5,20].

The incidence of *Aeromonas* in human infections worldwide is unknown, but a study in California (1988) showed that the annual incidence of *Aeromonas* infections was 10.5 cases per million people [4,151]. Janda and Abbott [4] also reported that in 2004 in England, the estimated incidence of *Aeromonas* bacteremia was 1.5 cases per million people and in France in 2006 it was estimated that it was 0.66 cases per million [152]. Another study conducted in Taiwan between 2008 and 2010 showed that the incidence of bacteremia by *Aeromonas* was 76 cases per one million individuals [153]. Those studies therefore show that the incidence of diseases caused by *Aeromonas* vary by geographical location and can be related to bad hygiene habits in undeveloped regions [3,5,152,153,154,155,156,157,158].

A literature review describing *A. dhakensis* and the molecular methods used for its identification revealed that the reported prevalence of the most predominant clinical species of *Aeromonas* has changed over the years [3,5]. This species was described after the reclassification of *A. aquariorum* and *A. hydrophila* subsp. *dhakensis.* Until then *A. caviae*, *A. veronii*, and *A. hydrophila* were considered to be the most prevalent clinical species [3,4,5,159]. However, based on the molecular identification of 817 strains obtained from different studies around the world, 94.7% of the strains associated to clinical cases correspond to four species: *A. caviae* (30.5%), *A. veronii* (23.1%), *A. dhakensis* (23.0%), and *A. hydrophila* (18.1%) [5]. 

An update to the data, adding 1034 strains in more recent studies, shows that those four mentioned species represent 95.4% (1766/1852) of the *Aeromonas* strains linked to human infections. Table 1 shows this new evaluation with a relative abundance of the species varying slightly, with 37.26% of the strains being *A. caviae*, 23.49% *A. veronii*, 21.54% *A. dhakensis*, and 13.07% *A. hydrophila.* However, the frequency of these species also varies by country. In fact, so far *A. dhakensis* is the most prevalent species in tropical climates (Malaysia, Taiwan, and Australia), with clinical importance in those countries [5,68,160,161]. The current confusion between *A. hydrophila* and *A. dhakensis* may have consequences, since the latter species is more virulent and shows greater cytotoxic activity [68].

Until now, only 16 species have been found in clinical samples [5], but two new species (*A. enterica* and *A. intestinalis*, in the process of description) isolated from human feces [31] are included in the list (Table 1). Nevertheless, the prevalence in clinical infections of the cryptic species *A. rivipollensis* [47], uncovered by *A. media*, is still not known [95].

One of the largest studies that has characterized clinical strains by molecular methods is the one performed by Latif-Eugenín [15] in her PhD thesis, who re-identified 422 isolates from Spanish hospitals using the sequences of the *rpoD* gene. The results showed that only 176 (41.6%) were originally correctly identified at species level. Of the 422 re-identified strains, 260 (61.6%) were identified as *A. caviae*, 91 (21.5%) as *A. veronii*, 27 (6.4%) as *A. hydrophila*, 19 (4.5%) as *A. media*, 5 (1.2%) as *A. dhakensis*, 2 (0.5%) as *A. allosaccharophila*, 2 (0.5%) as *A. salmonicida*, 1 (0.2%) as *A. bestiarum*, 2 (0.5%) as the new *Aeromonas* spp. mentioned above, and 13 (3.1%) did not belong to the genus *Aeromonas* at all. The results from that study differ from Figueras and Beaz-Hidalgo [5], and from the updated data shown in Table 1, which includes data from Latif-Eugenín [15]. In all cases, the most prevalent species was shown to be *A. caviae* (61.6% Spain vs. 37.26% in Table 1). *A. dhakensis* was less prevalent in Spain (1%) when compared with the the up-to-date reported data (21.54%). With all of the data taken together, 98% of the *Aeromonas* species identified in clinical cases belong to five species: *A. caviae*, *A. dhakensis*, *A. veronii*, *A. hydrophila*, and *A. media* (Table 1). Recently, Fernández-Bravo [20] demonstrated that human monocytes that showed the highest cell damage were coincidentally those that induced a higher expression of immune-related genes and were the most prevalent clinical species *A. dhakensis*, *A. veronii*, and *A. caviae.*

*Aeromonas* infections often involve more than one type of bacteria in the same clinical sample, which is defined as a polymicrobial or mixed infection [3,5,152,154,155,156,183]. Polymicrobial infections occur in 80% of cases of respiratory tract infections and cholangitis cases, and in 77% of cases of surgical site infections, followed by 60% of wound infections, and 30% of bacteremia [5,152,167,183]. The dominating bacteria in those mixed infections depends on the type of infection. In diarrhea cases, *Aeromonas* occur in combination with *Campylobacter* and *Salmonella*, while in wound infections, *Staphylococcus aureus* prevails [4]. Infections in which two *Aeromonas* strains were recovered from the same clinical sample, either two different strains of the same species or two different species, have occasionally been reported [183,184,185]; although, Mosser et al. [183] estimated a 5% incidence of dual or mixed infection. The latter authors suggested using *Caenorhabditis elegans* model of infection for the severity of the infections with more than one strain. Grim et al. [186] and Ponnusamy et al. [187] described complex and dynamic interactions of two strains of *A. hydrophila* that were isolated from an immunocompetent individual who developed necrotizing fasciitis. They suggested that the type six secretion system (T6SS) and exotoxin A (ExoA) were involved in this complex infection. Recently, using a mouse peritonitis and necrotizing fasciitis models, Fernández-Bravo et al. [188] used the mutants of T6SS and ExoA of these strains to elucidate the possible role in mixed infections and demonstrated that both virulence factors play a role after monomicrobial and polymicrobial infection.

### 4.5. Gastroenteritis

The first documented isolation of *Aeromonas* in feces dates from 1961, although *Aeromonas* was already isolated as a causative agent of myositis in a Jamaican woman in 1954 [189]. Several studies have associated *Aeromonas* with gastroenteritis [3,189,190,191,192,193,194,195]. Nausea, vomiting, fever, and abdominal cramps occur only in a fraction of patients, while colitis occurs in a third of diarrhea cases caused by *Aeromonas* [189]. The most frequent clinical presentation is secretory water, ranging from 75% to 89% of all cases in which this microorganism was the only associated pathogen [4,5]. Food and Drug Administration (FDA)-cleared multiplex molecular-based syndromic stool panels have replaced stool culture for bacterial pathogens in many laboratories. However, this commercial system does not detect *Aeromonas* [195].

Gastroenteritis caused by *Aeromonas* is a problem among the pediatric population, occuring mainly in children under three years with an incidence between 2.3% and 13% in countries like Taiwan and Nigeria, respectively [3,196]. However, studies by Bravo et al. [197] and Mansou et al. [194] showed a 7.5% and 1.4% incidence of *Aeromonas* children’s diarrhea, respectively, in Spain. Between 2–20% of gastroenteritis cases are monomicrobial and only 0–2% of children are asymptomatic carriers [198]; the diarrhea might last for one or two weeks and feces shows a liquid consistency [5,196].

The incidence in adults varies from 2% reported in Sweden, 6.9% in Hong Kong in healthy people, and 13% in immunocompromised patients [196]. Among patients in Spain with traveler’s diarrhea, *Aeromonas* was defined as a causative agent in 2% of cases [199], while in Japan it was isolated in 5.5% of cases, and 8.7% in Finland [196]. The infection was monomicrobial in 5.5% of the cases of traveler’s diarrhea [190,198,199].

Nowadays the enteropathogenic role of *Aeromonas* is clearly demonstrated in a study by Teunis and Figueras [192] using data derived from different human outbreaks [42,200,201,202,203]. It concluded that *Aeromonas* should be considered to be a human enteropathogen, much like *Campylobacter* and *Salmonella.* In addition, the same strain (genotype) that causes the diarrhea was isolated from water and food in several studies [82,119,120,204].

#### 4.5.1. Wound Infections

Wounds are the second most frequent route of entry of *Aeromonas* to humans after the oral–fecal route [4,5,196]. Infections caused by *Aeromonas* can occur on any skin or mucous surface, although the extremities are the most common sites [4,205]. Most cases affect healthy people and are often associated with traumatic events and burns and scolds related to water and soil [4,5,159]. In a retrospective study of 129 cases of skin and soft tissue infections in Taiwan attributed to *Aeromonas*, 78% of patients had suffered previous trauma, and in 30% of cases there was exposure to water [206]. Additionally, *Aeromonas* was the most isolated microorganism following natural disasters such as the tsunami in Thailand in 2001 (*Aeromonas* accounted for 22.6% of all isolates) and Hurricane Katrina in the southeastern United States (2005), mainly associated with wound infection [207,208].

Among the *Aeromonas* nosocomial infections, the most common is those associated with surgical procedures, the so-called surgical site infections (SSI). Tena et al. [32,167] reviewed a series of nine cases that occurred in Spain (eight in Guadalajara and one in Zaragoza) between 1990 and 2007, together with 15 cases cited in the literature, confirming that the majority were associated with extraction of the appendix, gallbladder, and colon. In addition, the use of leech therapy lead to *Aeromonas* infections in 7–20% of the treatments [5,209,210]. This is due to the fact that *Aeromonas* are symbionts of the leeches, enabling them to digest the blood [5].

Wound infections due to *Aeromonas* can progress severely to necrotizing fasciitis (NF), usually in immunocompromised patients [5,211]. NF is commonly known as a flesh-eating disease that can cause hypotension, fever, necrosis, and gangrene and can be a life-threating infection [156,186,187,188,211]. *A. hydrophila* is the *Aeromonas* species most frequently associated in reported NF cases and in some cases, water was considered the source of infection [156,186,211,212,213,214]. Three studies report the complex interactions, among multiple strains of *A. hydrophila* that were isolated from an immunocompetent young girl who developed NF resulting in amputation of almost all the extremities [186,187,188]. The NF case involved the exposure of an open wound to river water into which the girl had fallen, and was originally considered to be a monomicrobial infection [186]. However, it was later recognized that of the four strains recovered from the wound, only two were different strains of *A. hydrophila* (same species) and the strains were named NF1 and NF2 [186]. Additionally, after mixed and single infections, these studies showed that *exoA* gene and T6SS play a role in NF developed after monomicrobial and polymicrobial infection [186,187,188].

#### 4.5.2. Bacteremia/Septicemia

Several cases of bacteremia/septicemia due to *Aeromonas* are published [152,215,216,217,218]. The incidence of bacteremia can range from 0.12–3.3% and the mortality rate associated with *Aeromonas* bacteremia is about 30% [4,5]; although previous studies showed incidences of 25% [219,220]. Based on the data collected by Figueras [196] and Janda and Abbott [4], bacteremia associated with *Aeromonas* is preferably monomicrobial, acquired in the community, and mainly affecting males. Additionally, several studies demonstrated that the most prevalent species associated with blood infections were *A. caviae*, *A. veronii*, *A. dhakensis*, and *A. hydrophila* [5]. The underlying diseases found in cases of bacteremia/septicemia were most commonly malignancy, hepatobiliary disease, and diabetes [5,15,221]. Moreover, the most common symptoms associated with *Aeromonas* bacteremia according to Janda and Abbott [4] included fever (74–89%), jaundice (57%), abdominal pain (16–45%), septic shock (40–45%), and dyspnea (12–24%). That same study also classified the bacteremia in four groups based on the affected populations, the main one being immunocompromised individuals (>80%), followed by those who suffered a traumatic accident, then the cases that affect healthy people, and finally those that involve patients undergoing reconstructive surgery and/or leech therapy [4].

#### 4.5.3. Other Infections

Other infections with less incidence associated with *Aeromonas* have been reported: (i) respiratory tract infections; (ii) urinary tract infections, and (iii) spontaneous bacterial peritonitis (SBP) [4,5,196]. Although *Aeromonas* pneumonia is rare, some cases have been described [5,206,222,223]. Treatment with carbapenems is effective, although some cases of pneumonia have been fatal [223]. *Aeromonas* were also described as a causal agent in urinary tract infections [5,224,225]. The majority of the cases were reported in patients with various underlying diseases, such as spina bifida, bilateral renal dilatation, and diabetes mellitus. Lastly, SBP occurs in 16% of patients with cirrhosis [226].

## 5. Virulence Factors

*Aeromonas* infections present a wide variety of clinical manifestations and are considered multifactorial [18,19]. Various virulence factors enable *Aeromonas* to overcome the host immune response and can cause infections [5,18].

### 5.1. Structural Components

The adhesion of bacteria to host tissues is a critical step in the initial phase of infections by many microorganisms. Bacteria adhere to host tissues and cells and alter their defense mechanisms thereby initiating colonization. The most studied structural components in *Aeromonas* involved in the colonization process are the flagella, pili, capsule, S layer, and lipopolysaccharides (LPS) [18,19].

Flagella are organelles responsible for bacterial mobility composed of a filament, hook, and basal body. The genus *Aeromonas* has a polar and lateral flagellum [18,19]. The presence of lateral flagella gives the bacteria a fast or “swarming” type of mobility that allows them to move on solid surfaces and form biofilms. The polar scourge allows mobility by “swimming” in liquid environments [18,19,227].

Pili are filamentous structures found on the surface of bacteria, with subunits known as pilin. In addition to being an adhesion organelle, this structure is also involved in other functions, such as DNA transfer, biofilm formation, cell aggregation, and invasion of host cells [19].

The LPS have three subunits: the polysaccharide O (O antigen), the core of the LPS (central polysaccharide), and lipid A, which anchors them to the outer membrane of the bacteria [19,228,229]. These play an important role in the organization and maintenance of the outer membrane and are responsible for the virulence and producing a non-specific inflammatory response [17,19]. The toll-like receptors (TLRs), TLR4 in particular, are the best known membrane protein that recognizes LPS, which induces the activation of the immune response essential for an antibacterial defense [18,21].

Capsule is a polysaccharide structure that covers the outer membrane of bacteria involved that interacts with the environment. The capsule is described as an important virulence factor that is resistant to phagocytosis and the complement system [18,19,230].

The S layer is a surface protein layer of paracrystalline that is produced by a wide range of bacteria to form the outermost cell envelope [18,19]. In addition, this layeris associated with several functions related to pathogenicity.

### 5.2. Extracellular Proteins

The interaction between pathogenic bacteria like *Aeromonas* and host cells is produced as indicated by their extracellular components and toxins that are secreted into the extracellular space, such as proteases, lipases, enterotoxins, hemolysins, and Shiga toxins, among others [4,18,19,168,198,231,232,233,234].

Cytotonic and cytotoxic enterotoxins have been described in *Aeromonas* [18,19]. There are two groups of cytotonic enterotoxins: thermolabile (Alt) (56 °C for 10 min), which does not react with the cholera antitoxin, and thermostable (Ast) (100 °C for 30 min), which does react with the cholera antitoxin [198]. The cytotoxic enterotoxin (*act*) inhibits the phagocytic activity of host cells, produces hemolysis, and increases the levels of tumor necrosis factor α (TNF-α) and interleukin (IL-1β) in the RAW 264.7 murine macrophage cell line [235].

Two types of hemolysins are defined in *Aeromonas*, α and β, with physiological and functional differences [236] but which are capable of forming pores in the membrane of the target cell generating their osmotic lysis [231]. Aerolysin is the prototype hemolysin of the genus, encoded by the *aerA* gene [19]. It was characterized in 1974 by Bernheimer and Avigad [237]. Aerolysin is secreted by the type two secretion system (T2SS), which is sec-dependent, transcribed as a pre-pro-aerolysin that undergoes various processes of maturation during its secretion to become an active 47.5 kDa aerolysin in the extracellular environment [15].

Proteases allow *Aeromonas* to persist in several habitats and facilitates interactions with other microorganisms. In general, proteases contribute to pathogenicity as they promote invasion by direct damage to host tissue or by proteolytic activation of toxins [18,19]. Additionally, they can also contribute to the establishment of an infection that exceeds the host initial defenses, inactivating the complement system or providing nutrients for cell proliferation [19]. *Aeromonas* produces at least three types of proteases: metalloprotease (*ahp*, *aphB*), acetylcholinesterase, and serine protease (*aspA*) [84,88].

*Aeromonas*, like many other pathogenic bacteria, secrete lipases to the medium that acts as hydrolases on membrane lipids [18,19]. These can provide nutrients or constitute virulence factors when interacting with human leukocytes or by affecting various functions of the immune system [18]. An important lipase in the genus *Aeromonas* is glycerolphospholipid: cholesterol acyltransferases (GCAT), which have the ability to digest the membranes of erythrocytes and produce their lysis [19]. In addition, *gcat* gene can be used for identification at genus level [60,112].

According with the literature, *A. hydrophila* expresses various degradative enzymes that can contribute to its virulence, among which are collagenase, elastase, and enolase. Collagenase was shown to have cytotoxicity in Vero cells [238]. Enolase, a glycolytic enzyme secreted and expressed on the surface, was identified as a virulence factor in *A. dhakensis* SSU, which was observed to have functions in the degradation of blood plasma proteins [239].

The function of the Shiga toxin is the inactivation of ribosomes (arrest of protein synthesis) of vascular endothelial cells, leading to cell death [19,168]. They are also encoded in bacteriophages that are normally integrated in the bacterial chromosome. When the lytic cycle is induced, large numbers of them capable of infecting other bacteria are released, acting as horizontal transmission vectors of the *stx* genes [240]. Recently, Shiga toxins have only been detected in strains of *Aeromonas* recovered from food [234].

### 5.3. Secretion Systems

Nowadays, six secretion systems have been described in Gram-negative bacteria, all of which are involved in the transport of virulence factors to the extracellular medium or directly into the host cell. The T2SS and type five secretion system (T5SS) are Sec or Tat dependent, which means that virulence factors secreted by these mechanisms contain signal peptides that are recognized by Sec and Tat proteins allowing their translocation, while type one secretion system (T1SS), T3SS, type four secretion system (T4SS) and T6SS are Sec independent, so they transport their proteins directly to the cell surface or host cell [241].

The T2SS is an exclusive secretion system of proteobacteria that shows a common evolutionary origin with type IV pili [19,241,242]. This secretion system is essential in the pathogenesis of various microorganisms such as *Vibrio cholerae*, *Legionella pneumophila*, *Escherichia coli* [242], *A. hydrophila* [84], and *A. salmonicida* [88].

The T3SS or injectosome is one of the secretion systems by which proteins can be injected directly from the bacterial cell protoplasm to the cytoplasm of the target cell or to the extracellular space [18,19,241]. In *Aeromonas*, T3SS genes have been extensively studied: AexT (ADP-ribolisant toxin), AopU, which inhibits the nuclear Kappa B factor involved in the activation of IkB (protein kinase), AopH (tyrosine phosphatase), AopO (serine/threonine kinase), and AexU (similar to AexT toxin) [17,19,243,244,245,246,247,248,249,250]. Studies of infections by *A. salmonicida* and *A. hydrophila* strains mutated for T3SS have shown that that they have a lower virulence than the non-mutated strains [17,247,248,249,251]. Recently, 21 *Aeromonas* T3SS likely effector families were described using bioinformatics tools with the experimental analysis, which will be useful in future research to identify bacterial effector proteins in other genera [252].

The T4SS is the only secretion system capable of transporting DNA as well as proteins, which gives the bacterial conjugation system a homologous role [241]. This secretion system plays a crucial role in the propagation of resistance genes and virulence [18,19].

The T6SS or Vas (virulence-associated secretion) was the last secretion system to be recognized in 2006 [253]. Like the T3SS and T4SS it is able to inject protein effectors directly into the cytosol of the target cell, although this secretion system has been identified in non-pathogenic organisms or symbionts [253]. It appears to be highly conserved and can be found in one or more copies in diverse Gram-negative species, such as *Vibrio cholerae*, *Pseudomonas aeruginosa*, *Yersinia pestis*, *E. coli*, and *Salmonella enterica*, among others [18,19,84]. Among the secreted effector proteins, the best known are G repeat proteins (VgrG) and hemolysin-co-regulated protein (Hcp). In *Aeromonas*, it was first detected in 2006, following the sequencing of the complete genome of *A. hydrophila* [19,84], although its functionality was unknown. Suárez et al. [254] demonstrated the functional role in virulence of this secretion system in *A. hydrophila.* In recent years, T6SS has been reported to have an additional “antibacterial” role in polymicrobial infections, eliminating competitor bacteria [255]. In fact, Carruthers et al. [256] suggested that T6SS of *Acinetobacter baumanii* plays a role in the competence with other bacteria. In the same way, a different study demonstrated that the T6SS of *Shigella sonnei* confers an advantage to this species when it competes with *Shigella flexneri* and *E. coli* and this advantage is reduced in T6SS defective mutants [257]. Recently, the T6SS was associated with a role in the mixed infections by two *A. hydrophila* strains (NF1-NF2) recovered from a case of necrotizing fasciitis [187,188]. The data suggested that the expression of the effectors related to T6SS might be differentially regulated in both strains, causing a different progression of the disease following mixed infections from progression following a single infection [187,188].

### 5.4. Quorum Sensing

Quorum sensing (QS) it is a mechanism for regulating genetic expression in response to cell population density [19]. The cells involved produce and excrete substances, called autoinductors, which serve as a chemical signal to induce collective genetic expression. [258]. The “signal” molecule in Gram-negative bacteria is N-acyl homoserine-lactone (AHL) and in *Aeromonas*, the AHL is able to modulate the host immune response [19,186,259]. Several studies have shown the importance of AHLs in regulating a range of biological functions such as biofilm formation, antibiotic production, and warming motility and the different QS systems harbor differences in their influences [260,261,262,263,264,265,266]. Chan et al. [260] found in the whole genome of the strain *A. veronii* 159 sequences of QS-related genes, providing a model for exploring it roles in virulence and as a potential target for *Aeromonas* treatment. Talagrand-Reboul et al. [266] focused their review on the knowledge of the biofilm and QS in *Aeromonas*, explaining that biofilm provides a high cell-density that allows interaction between bacteria with the QS systems in *Aeromonas.* That study also reported the possible role of this biofilm formation in mixed infections and should be further studied. Recently, Liu et al. [264] showed that the infection with the asal-mutant (failed to produce the short chain AHLs signal) in a marine fish isolated of *A. salmonicida* affected the biofilm formation. Blöcher et al. [261]. demonstrated the development of anti-QS compounds to overcome the resistant bacteria strain *A. caviae* Sch3, inhibiting biofilm formation. That pilot study contributed to finding new therapies to overcome the bacterial antibiotic resistance problem. Ding et al. [262,263] demonstrated the impact of curcumin liposomes against *A. sobria* and *A. hydrophila* in biofilm formation, due to the anti-QS properties of this dietary supplement. This information might be useful to design QS inhibitors. The same occurs with tannic acid, a potent quorum quencher that regulates the biofilm formation of *A. hydrophila* [265].

### 5.5. Metal Ions

Metal ions are essential for the correct functioning of microorganisms biological process, “metallostasis” [267]. They also play an important role in the host–pathogen interaction [268]. In the course of an infection, the host restricts the accessibility of crucial metals, by inactivating the metal-dependent processes of the bacterial pathogen that compensates this limitation by generating different proteins [268,269,270].

The mechanisms for iron acquisition are known to play an important role in the development of the infection [19]. Two mechanisms are described so far: siderophore-dependent and siderophore-independent. Siderophores are peptides that present a functional group with an affinity to iron ions that need specific cell membrane-bound receptors and a cell-associated apparatus to incorporate the metal into the bacterial metabolism. The siderophore-independent mechanism consists of a bacterial outer membrane protein that binds host-specific iron. In addition, the gene expression related to the iron acquisition is regulated by protein Fur [18,19].

Other metal cofactors with a role in the pathogenicity of *Aeromonas* are also described, for example, copper and silver resistance genes coding for different proteins [271,272]. Recently, Fernández-Bravo et al. [273] demonstrated that the nickel-binding protein HypA in *Aeromonas* might play a role in acid tolerance and in the defense against macrophages.

## 6. Host–Pathogen Interaction

**Figure 2:**
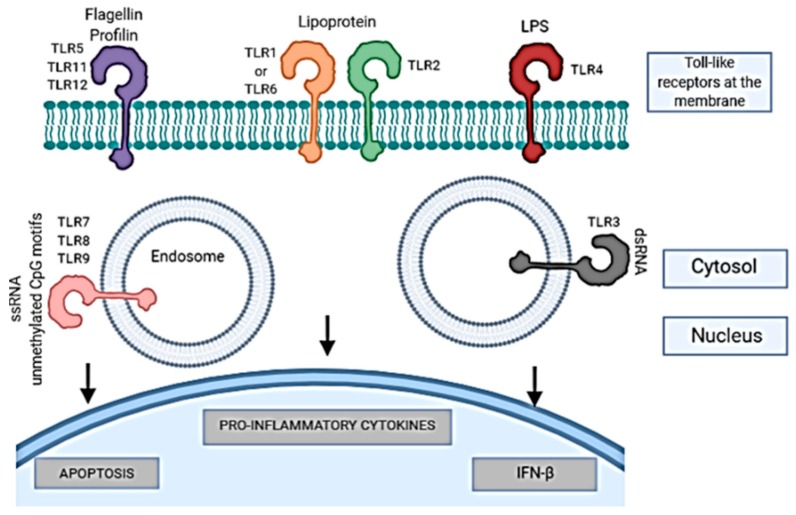
Toll-like receptors (TLRs) pathways that induce the inflammatory response mediated by cytokines (adapted from [278]). Created with Biorender.

**Figure 3:**
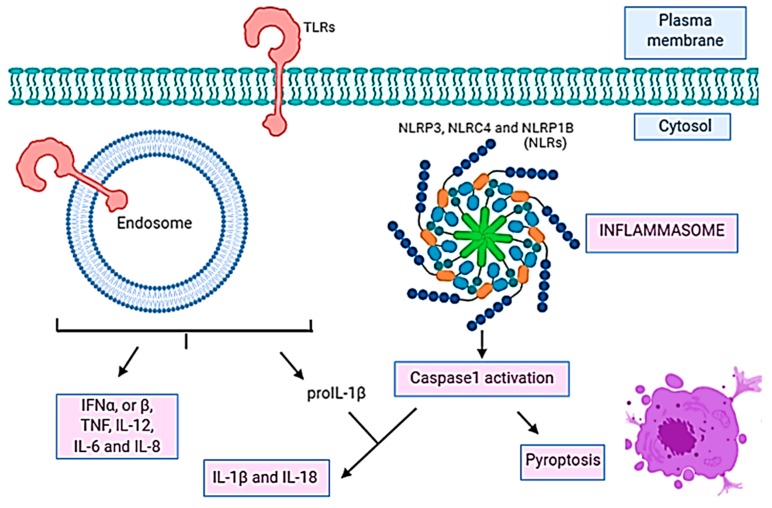
Pyroptosis pathway with Toll-like receptors (TLRs) and Nod-like receptors (NLRs) acting in concert (adapted from [275]). Created with Biorender.

The host–pathogen interaction activates an immune response as a result of antigen exposure. Two types have been described: innate and adaptative [274]. The innate immune response is activated after recognition of structures associated with microbes named pathogen-associated molecular patterns (PAMPs) by receptors named pattern recognition receptors (PRRs) present and expressed in a variety of cells, like neutrophils, monocytes or macrophages [275,276,277]. The PRRs include TLR members, nucleotide binding, oligomerization domain-containing receptors (NOD-like receptors (NLRs)), retinoic acid-inducible gene (RIG) I-like RNA helicases, C-type lectins, and AIM2-like receptors (ALRs). The TLRs are membrane PRRs that induce the phagocytosis of the pathogen and activate the expression of cytokines in the host, initiating the inflammatory response [278]. It was also demonstrated that the TLRs recognition results in the induction of apoptosis [279] (Figure 2). NLRs are cytosolic PRRs, which activate a different type of cell death than apoptosis (programmed cell death) which is caspase-1 dependent and named pyroptosis [280,281] (Figure 3).

The expression of several TLR genes (TLR1, TLR2, TLR3, etc.) following *Aeromonas* infection has been described in the last years, as shown in Table 2. Two recent studies analyzed the expression of different TLRs following an *A. hydrophila* infection using a fish model [282,283]. The data obtained in these studies demonstrated that the immune response presented tissue-specific patterns. However, the higher expression of TLRs indicated that they play an important role in the innate response against *A. hydrophila* [282,283]. TLR4 is a transmembrane receptor of monocytes, macrophages, and dendritic cells that senses molecules such as LPS present in the cell walls of Gram-negative bacteria [284,285]. Previous studies investigated the TLR4 signaling pathway after *A. hydrophila* infection by using a fish model, indicating the importance of this receptor in increasing the innate immunity response to bacteria invasion. Data from two studies showed that the expression of TLR4 might be induced following an *A. hydrophila* infection in a minnow *Gobiocypris rarus* and the blunt snout bream *Megalobrama amblycephala* [21,286]. Similarly, an interesting study was conducted by Srivastava et al. [24] using zebrafish as a model.

Gene expression of immune response mediators such as cytokines, tumor necrosis factor alpha and beta (TNF-α, TNF-β), interferon gamma (IFN-γ), interleukin 6, 8, and 10 (IL-6, IL-8, IL-10) among others, as well as chemokines, such as C-C ligand 3 (CCL3) induced by TLR signaling have been studied, as summarized in Table 2 [290,294,296,306]. Studies in which *A. salmonicida* was used to infect rainbow trout showed an upregulation of TNF-α among other cytokine genes, involved in systemic inflammation [290,306]. However, differences in expression were observed based on the tissue studied, such as the intestine or kidney [290,306]. A recent study by Kong et al. [294] established that *A. hydrophila* might induce an overexpression of the pro-inflammatory cytokine gene TNF-α in the intestine of fish, deteriorating the integrity of the mucosal barrier structure. Similarly, the expression of different chemokines, which are a family of small cytokines with an important role in the immune response, has been studied after *A. hydrophila* infection in eel and grass carp [297,298].

There are various studies on the expression of cytokines and chemokines after *A. hydrophila* infection using mice and several cell lines, such as murine macrophage from blood (RAW 264.7), human colon cancer cell line (HT-29), and a cancerous cell line (HeLa) (Table 2). Those studies, demonstrated, for instance, an alteration in the production of cytokines and chemokines after infection. In another study, the possible protective role of QS was evaluated using mice treated with AHL before infection by *A. hydrophila* [259]. Results showed a reduced level of cytokines and chemokines as well as of the bacterial load in the organs. This work suggested that the AHL pre-treatment modulated the innate immune response in mice and increased the survival of the mice following an *A. hydrophila* infection [259].

Table 2 shows that several studies reported that apoptosis can be induced in vitro following an *Aeromonas* infection, mainly with *A. hydrophila*, using different cell lines, such as RAW 264.7, HeLa, HT-29 or mouse BALB/C monocyte macrophage (J744). Other studies have also demonstrated the capacity of *A. hydrophila* and *A. veronii* to cause apoptosis in kidney leukocytes or head kidney macrophages obtained from fish. In relation to proteins associated with apoptosis, TP53 is a suppressor tumor protein with an important role in the programmed cell death, whose expression was studied in zebrafish after *A. hydrophila* infection [302]. The induction of apoptosis was also studied by evaluating the caspase 3 protein (CASP3) activation in HeLa cells [295]. The results from the study demonstrated that the AexU gene in *A. hydrophila* induced the apoptosis via CASP3 [295].

The inflammasome consists of a complex of caspase-1, PYCARD domain (ASC), AIM-2, and the NLR receptor. NLR subset inflammasomes, such as NLR pyrin domain containing 3 (NLRP3), NLR card domain containing 4 (NLRC4) or NLR pyrin domain containing 1 (NLRP1), promote the maturation and secretion of the pro-inflammatory cytokines, interleukin 1 beta (IL-1β) and IL-18, resulting in pyroptosis. Following an *A. hydrophila* infection, the activation of caspase-1, and the release of interleukin 1 beta (IL-1β), followed by the cell death called pyroptosis have been studied using macrophages as a model [23]. The results suggested that *A. hydrophila* induces an inflammatory response via NLRP3 inflammasome, which comprises the NLR protein (NLRP3), the adapter ASC, and caspase-1 [23]. Another study by the same Japanese group demonstrated that inflammasomes-mediated caspase-1 activation (NLRP3 and NLRC4) is involved in host defenses against systemic *A. veronii* infection in mice and macrophages [22,290,306] (Table 2).

The nuclear factor kappa-light-chain-enhancer of activated B cells (NF-κB) is a protein complex that plays a role in the regulation of the immune response, controlling the pro-inflammatory cytokines after the stimulation of the TLRs. RelA is a protein involved in NF-κB formation and studies using fish as a model established that the overexpression of the RelA following *A. hydrophila* infections induced the production of several pro-inflammatory cytokines (Table 2). c-Jun is an immune modulation protein that along with c-Fos, forms the AP-1 that is also an activator of pro-inflammatory genes such as TNF-α, IL-8, and IL-6, among others. However, it has been reported that when the c-Fos acts alone, this protein induces an increase in anti-inflammatory genes such as IL-10 or IL-4, among others [307]. These genes have been studied after an infection of human epithelial colorectal adenocarcinoma (Caco-2) cells with *Aeromonas* observing a possible role of these transcription factors in virulence [299] (Table 2).

## 7. Pathogenicity Studies

### 7.1. In Vitro Studies

Many studies of pathogenesis and virulence in *Aeromonas* have been carried out in recent years using in vitro cell lines (Table 3). The ability of *Aeromonas* to adhere, invade, and produce cytotoxicity has been defined, mainly following *A. hydrophila* and *A. caviae* infections, using human larynx carcinoma (HEp-2) and human Caucasian colon adenocarcinoma (Caco-2) cells [289,308,309,310]. Ghatak et al. [309] analyzed the cytotoxicity of 55 strains of *Aeromonas* using four cell lines, African green monkey kidney (Vero), Madin Darby bovine kidney (MDBK), baby hamster kidney (BHK-21), and lymphoblast B mono lymphoblast (B95a) cells. The data obtained from that study suggested that the Vero cell line is the best model for examining and testing the cytotoxicity of *Aeromonas* species [309]. In 2012, Krzyminska et al. [310] demonstrated that the presence of T3SS in the *Aeromonas* strain tested was able to mediate cell-contact cytotoxicity, destruction of host epithelial cells, and tissue damage in different cell lines: Chinese hamster ovary (CHO), HEp-2, and Vero.

Moreover, Merino et al. [289] demonstrated by the generation of mutants that the flagella glycosylation in *A. hydrophila* plays an extremely important role in the adhesion to Hep-2 cells and in biofilm formation. In a recent study, Dos Santos et al. [308] evaluated the capacity of *Aeromonas* species to adhere, invade, survive, and produce cytotoxicity through the use of three different cell lines: HEp-2, Caco-2, and human colorectal adenocarcinoma (T-84). The results suggested that cytotoxicity and adhesion would depend on the type of cell line analyzed, indicating that the majority of *Aeromonas* strains survived in the T-84 cells.

Regarding to polymicrobial infections, Ponnusamy et al. [187] studied the mixed infections by two strains of *A. hydrophila* (NF1-NF2) using the murine macrophages cell line RAW 264.7. The data obtained suggested that there was a different course of the disease after mixed infection compared to the single infection.

### 7.2. Animal Models

Which experimental animal model to use is one of the most important questions in the study of virulence [17]. Recreating *Aeromonas* infections in different animal models has been the feature of some studies, and Table 4 describes the animal models used and the latest relevant studies.

In many studies, mice are considered a good model for studying pathogenicity in *Aeromonas*, because mice are susceptible to a similar range of microbial infections as humans [17,325]. In three recent studies by Grim et al. [186], Ponnusamy et al. [187], and Fernández-Bravo et al. [188] regarding the progression of NF, the role of the two strains of *A. hydrophila* involved in a mixed infection was studied using mice as the model of infection. In addition, strains were marked with a bioluminescent gene to elucidate dissemination into the peripheral organs. Another interesting study with mice was carried out by Chen et al. [315], who compared the pathogenic capacity of *A. hydrophila*, *A. dhakensis*, *A. caviae*, and *A. veronii* by the analysis of survival rates using BALB/c mice. Results suggested a variation among the most clinically prevalent species, with *A. dhakensis* being more virulent than *A. hydrophila*.

In 2016, Romero et al. [17] suggested that zebrafish larvae could be used as the host model to assess the virulence of *A. hydrophila*, which would enable us to stop using mice. Zebrafish were used in another study as a model of *Aeromonas* species co-infection [293], and showed a higher mortality rate under co-infection in relation to the single infection. Additionally, zebrafish were used to study the immune response against *Aeromonas* [24].

Recently, the catfish was used as a fish model to clarify the pathogenic mechanisms in *A. hydrophila* [317], the blocking of aerolysin activity after *A. hydrophila* infections [316], and the enterotoxic effects after *A. hydrophila* infection [318], among others.

Chen et al. [324] evaluated the nematode of *C. elegans* as an animal model for studying *Aeromonas* infections, suggesting that *A. dhakensis* was the most virulent species in comparison with the other most prevalent clinical species. In 2016, Chen et al. [323] also used *C. elegans* as a disease model for muscle necrosis following an *A. dhakensis* infection. In relation with the mixed infections, Mosser et al. [183] also used the *C. elegans* model and demonstrated that the virulence of one *Aeromonas* strain could be higher in the presence of another strain, so the combination of both strains caused a higher mortality rate of the nematodes than a single infection.

Other interesting animal models of infection have also been proposed, but less often studied in *Aeromonas*: (i) blue gourami was used to study the role of an endogenous serum lectin in the immune protection against *A. hydrophila* infections [320], as well as for the identification of putative virulence factors in *A. hydrophila* strains [321]; (ii) *Dictyostelium amoebae* as an alternative host model for evaluating the virulence of *Aeromonas* [322].

## 8. Antimicrobial Resistance

Resistance to antimicrobial agents is a genetic–evolutionary response mediated by the presence of genes, some of which are found in plasmids, integrons or in the genome of the bacteria. Except for a few strains and the species *A. trota*, *Aeromonas* are described as resistant to ampicillin [1,4,42]. These bacteria are also resistant to other penicillins and first-generation cephalosporins [4,199,326]. However, *Aeromonas* are susceptible to monobactams, carbapenems, third- and fourth-generation cephalosporins, aminoglycosides, and fluoroquinolones [327].

Ndi and Barton [328] reported an increase in resistance to beta-lactam antimicrobials (penicillins and derivatives, cephalosporines, carbapenems, and monobactams) by the presence of genes that code for the production of beta-lactamases [4]. Three mainly β-lactamases were described in *Aeromonas*: class B metallo-β-lactamase, class C cephalosporinase, and class D penicillinase [329]. Fosse et al. [330] characterized the β-lactamases associated with the different *Aeromonas* species in five groups (i–v): (i) *A. hydrophila* complex strains expressing class B, C, and D β-lactamases; (ii) *A. caviae* strains expressing class C and D β-lactamases; (iii) *A. veronii* strains containing class B and D lactamases; (iv) *A. schubertii* strains harboring class D lactamases; and (v) *A. trota* strains with class C β-lactamases. It also appears that many *A. veronii* bv. sobria isolates also produce a class C cephalosporinase [4]. In fact, Sanchez-Cespedes et al. [331] reported a case in which the patient developed cholangitis and imipenem resistance in an *A. veronii* biovar *sobria* infection, probably as result of prior antibiotic treatment.

Recently, the studies of antimicrobial profiles in *Aeromonas* has increased, due to the necessity of responsible use of antibiotics [181,182,332,333,334,335,336,337]. Wimalasena et al. [337] suggested that in relation to animals, pet turtles could be a health risk to humans, due to the antimicrobial resistant *Aeromonas* strains found in these animals. Dias et al. [332], also reported that wild animals are considered a potential public health risk because they are potential reservoirs of resistant strains of *Aeromonas.* Previous studies comparing clinical isolates demonstrated that the resistance genes were species- and isolation site-dependent. The resistant strains were mostly detected from peritoneal fluids [181]. In addition, Zhou et al. [182] demonstrated that clinical strains isolated from extra-intestinal infections were resistant to several antibiotics, however, third-generation cephalosphorins, fluoroquinolones, and aminoglycosides could be an option to treat these infections.

Data from other studies agree that resistance could be related to mobile genetic elements such as plasmids, insertion elements, pathogenicity islands or cassettes associated with integrons [338,339,340]. Vega-Sánchez et al. [341] showed the incidence of class 1 integron and β-lactamases genes in *Aeromonas* isolates from rainbow trout. In 2017, Pitowska et al. [342] studied the variety of beta-lactamases genes present in *Aeromonas* spp. isolated from wastewater, finding a high number of genes encoded by plasmids. They suggest that *Aeromonas* antibiotic resistance strains could disseminate from wastewater to other environments. Recently, Hossain et al. [334] observed a higher incidence of different antimicrobial resistance genes and class 1 integron gene cassettes in resistant *Aeromonas* isolates. That study was conducted to prevent the consequences of inappropriate antimicrobial use.

## 9. Conclusions

Many questions were addressed in this review. However, *Aeromonas* is an important microorganism of the aquatic system, widely distributed in the environment, and one that colonizes and can infect humans and animals. *Aeromonas* infections will remain a health problem in the near future, considering the increased life expectancy of humans which will result in more elderly persons with potential underlying diseases, who are more susceptible.

Although we have extensive knowledge about the genus, different questions are arising pertaining to such things as potential new species, unusual resistance mechanisms, and mixed infections with different course of diseases. Based on this, it is important to continue the studies in order to provide more precise answers to these questions.

## Figures and Tables

**Table 1:** Distribution of *Aeromonas* spp. identified with molecular methods and recovered from different sites in association with human infections ^a^.

Species	Faeces	Wound	Blood	Respiratory Tract ^b^	Urine	Peritoneal Dialysate	Bilis	Ascitic	Abscess	Other ^c^ Fluid	Total
***A. caviae***	446	43	147	11	12	11	11	2	3	4	690 (37.26)
***A. veronii***	231	49	125	12	1	1	6	7	1	2	435 (23.49)
***A. dhakensis*** **^d^**	111	133	111	4	6	11	11	2	1	9	399 (21.54)
***A. hydrophila***	69	96	45	9	5	8	3	1	2	4	242 (13.07)
***A. media***	32	3	6		1						42 (2.27)
***A. trota***	2	2					1			1	6 (0.27)
***A. taiwanensis***	2	3	1			1					7 (0.32)
***A. salmonicida***	1	2							1		4 (0.21)
***A. jandaei***	3	2	1		1					1	8 (0.43)
***A. sanarellii***	1	4									5 (0.27)
***A. allosaccharophila***	2		1								3 (0.16)
***A. tecta***	2										2 (0.11)
***A. diversa***		2									2 (0.11)
***A. schubertii***		1	1								2 (0.11)
***A. bestiarum***	1	1	1	1							4 (0.21)
***A. popoffii***					1						1 (0.05)
***A. intestinalis*^e^**	1										1 (0.05)
***A. enterica*** **^e^**	1										1 (0.05)
**Total**	905	341	439	37	27	32	32	12	8	20	1852

^a^ Adapted from Figueras and Beaz-Hidalgo [5] and Latif-Eugenín [15] that included data obtained from different studies [38,54,66,68,89,144,153,160,161,162,163,164,165,166,167,168,169,170,171,172,173,174] and the results of 76 unpublished fecal isolates, with the addition of 865 new strains from several studies [15,175,176,177,178,179,180,181,182]. ^b^ 18 sputum strains; ^c^ Other includes: *A. caviae* (*n* = 4) of: vagina (3) and appendix (1); *A. veronii* (*n* = 2): sterile site (1) and ear fluid (1); *A. dhakensis* (*n* = 9): sterile site (6), joint fluid (1), eye (1), and bone (1); *A. hydrophila* (*n* = 4): ulcer exudate (1) and sterile site (3); *A. trota* (*n* = 1): cerebrospinal fluid (1); *A. jandaei* (*n* = 1): eye (1); ^d^ In many of the studies *A. aquariorum* was the name used for *A. dhakensis*; ^e^ Two new species of *Aeromonas* in the process of description.

**Table 2:** Studies (2009–2019) that evaluate proteins and processes involved in the immune response of the animal models or cell line host after *Aeromonas* spp. (adapted from [20]).

Proteins or Process Studied	Host	*Aeromonas* sp.	Reference
**Toll-like receptors (TLRs)** (TLR1, TLR2, TLR3, TLR4, TLR5, TLR7, TLR8, TLR9, TLR18, TLR19, TLR20, TLR22)	Fish, cells (EPC, HKLs, Hep-2)	*A. hydrophila*, *A. salmonicida*, *A. veronii*	[21,24,282,283,286,287,288,289,290,291,292]
**Cytokines** (TNF-α, TNF- β, IFN-γ, IL-6, IL-8, IL-10)	Fish, mice, cells (Hep-2, HKLs, RAW 264.7, HeLa)	*A. hydrophila*, *A. salmonicida*, *A. dhakensis*, *A. veronii/A. hydrophila*	[259,289,290,293,294,295,296]
**Chemokines** (CC-Chem14, CsCC-Chem20, CsCC-Chem25, CCL3, CCL5)	Fish, mice, cells (HeLa, RAW 264.7)	*A. hydrophila*, *A. dhakensis*	[259,295,297,298]
**Transcription factors** (JUN, RELA)	Fish, cells (Caco-2)	*A. hydrophila*, *A. salmonicida*, *A. veronii*, *A. bestiarum*, *A. allosaccharophila*	[299,300]
**Apoptosis** (TP53, CASP3)	Fish, mice, cells (HeLa, RAW 264.7, macrophages from fish, SLs, HKLs)	*A. hydrophila*, *A. veronii*	[295,301,302,303,304,305]
**Pyroptosis** (NLRP3, NLRC4, IL-1β)	Mice, macrophages from mice	*A. hydrophila*, *A. veronii*	[22,23]

Epithelioma papulosum cyprini cell line (EPC); head kidney leukocytes (HKLs), human epithelial cell line (Hep-2); murine macrophage cell line (RAW 264.7); human epithelial type cell line (HeLa); human epithelial colorectal adenocarcinoma cell line (Caco-2); spleen leukocytes (SLs). The strains of these studies [282,283,286,292,293,294,296,297,302] were identified by non-molecular methods or lack information about the identification.

**Table 3:** Studies (2009–2019) that investigate the virulence and pathogenicity of *Aeromonas* spp. using different cell lines (adapted from [20]).

Cell Line	Study	*Aeromonas*	Reference
**HEp-2, Vero**	Adhesion and cytotoxicity	*A. hydrophila. A. salmonicida*, *A. veronii*, *A. bestiarum*, *A. schuberti*, *A. eucrenophila*, *A. encheleia*, *A. jandaei*, *A. sobria*, *A. caviae*, *A. trota*, *A. media*	[311]
**HEp-2, CHO**	Cell-contact cytotoxicity	*A. hydrophila*, *A. caviae*, *A. veronii*	[310] ^a^
**C2C12**	Cytotoxicity	*A. hydrophila*, *A. dhakensis*	[68]
**HEp-2**	Adhesion, biofilm formation, and immune stimulation	*A. hydrophila*	[289]
**HEp-2, Caco-2,** **T-84**	Adhesion, invasion, and cytotoxicity	*A. hydrophila*, *A. caviae*	[308] ^a^
**RAW 264.7**	Role of mixed infections in Necrotizing fasciitis	*A. hydrophila*	[187]
**Caco-2**	Adhesion and cytopathic effect	*A. hydrophila*, *A. dhakensis*, *A. bestiarum*, *A. piscicola*, *A. salmonicida*	[312]
**HepG2, WLR-68**	Cytotoxicity effect of metalloprotease	*A. hydrophila*	[313] ^a^
**Caco-2**	Adhesion, invasion, and cytotoxicity	*A. salmonicida*	[314]
**J744.1**	Role of metallochaperone HypA	*A. hydrophila*	[273]
**RAW 264.7**	Role of mixed infections in Necrotizing fasciitis	*A. hydrophila*	[188]

Human epithelial cell line (HEp-2), kidney epithelial cells from African green monkey (Vero), Chinese hamster ovary cell line (CHO), mouse myoblast cell line (C2C12), human epithelial colorectal adenocarcinoma cell line (Caco-2), human colorectal adenocarcinoma cell line (T-84), murine macrophages cell line (RAW 264.7), hepatic cell line (WLR-68). ^a^ The strains were identified by non-molecular methods (RFLP, phenotypic characteristics, virulence factors).

**Table 4:** Relevant studies (2000–2001) of virulence and pathogenicity after *Aeromonas* infection using different animal models (adapted from [20]).

Model	Study	*Aeromonas*	Reference
**Mice** (*Mus musculus*)	Virulence factors, role of mixed infections in necrotizing fasciitis	*A. hydrophila*, *A. dhakensis*, *A. caviae*, *A. veronii*, *A. salmonicida*	[16,17,186,187,188,315]
**Catfish** (*Clarias gariepinus*, *Ictalurus punctatus*, *Hypophthalmichthys molitrix*)	Enterotoxic effects, virulence mechanism, transcriptome, aerolysin activity	*A. hydrophila*, *A. veronii*	[316,317,318,319]
**Blue gourami** (*Trichogaster trichopterus*)	Septicemia, immune responses	*A. hydrophila*	[320,321]
**Zebrafish** (*Danio rerio*)	Immune response, role of mixed infections in the virulence	*A. hydrophila/A. veronii* coinfection, *A. hydrophila*	[17,24,293]
**Slime mold** (*Trichogaster tricopterus*)	Pathogenicity	*A. salmonicida*, *A. hydrophila*	[322]
**Nematode** (*Caenorhabditis elegans*)	Virulence, immune response, necrosis	*A. hydrophila*, *A. dhakensis*, *A. veronii*, *A*, *caviae*	[183,323,324]

The strains of these studies [293,316,320,321,322] were identified by non-molecular methods or lack of information about the identification.
